# Wearable Nail Deformation Sensing for Behavioral and Biomechanical Monitoring and Human-Computer Interaction

**DOI:** 10.1038/s41598-018-36834-x

**Published:** 2018-12-21

**Authors:** Katsuyuki Sakuma, Avner Abrami, Gaddi Blumrosen, Stanislav Lukashov, Rajeev Narayanan, Joseph W. Ligman, Vittorio Caggiano, Stephen J. Heisig

**Affiliations:** IBM Thomas J. Watson Research Center, 1101 Kitchawan Road, Yorktown Heights, NY 10598 USA

## Abstract

The dynamics of the human fingertip enable haptic sensing and the ability to manipulate objects in the environment. Here we describe a wearable strain sensor, associated electronics, and software to detect and interpret the kinematics of deformation in human fingernails. Differential forces exerted by fingertip pulp, rugged connections to the musculoskeletal system and physical contact with the free edge of the nail plate itself cause fingernail deformation. We quantify nail warpage on the order of microns in the longitudinal and lateral axes with a set of strain gauges attached to the nail. The wearable device transmits raw deformation data to an off-finger device for interpretation. Simple motions, gestures, finger-writing, grip strength, and activation time, as well as more complex idioms consisting of multiple grips, are identified and quantified. We demonstrate the use of this technology as a human-computer interface, clinical feature generator, and means to characterize workplace tasks.

## Introduction

All known, extant primate species possess a flat fingernail if only on the hallux^1^. This feature is one of the few characteristics unique to the order Primates. The most recent common primate ancestor is estimated to have lived near the end of the Jurassic and the start of the Paleocene eras, approximately 65 million years ago^2^. The structure of the flat nail has been conserved this long testifying to its usefulness across many tasks and environments. Nails and volar pads are part of the integumentary system which covers and protects the body^3^. These structures interface with the environment and are used to both sense and manipulate. The apical volar pads of the fingers are also part of the somatosensory system, providing fine touch tactile feedback as well as haptic perception. The volar pads of the hands are specialized to provide a tough, but flexible gripping surface. These structures are used by humans in gripping thousands of times a day^4^, for social touching, dressing, grooming, scratching, food preparation, and eating. Every kind of tool use from teeth brushing, to typing, to driving a car involves grip. Although in most tasks the volar pads are in direct contact with the object, nails play an essential function to enhance both gripping and sensing capability by focusing the volar pulp on the object being manipulated^5^. Also, the nail plate itself can function as a tool for scratching, picking, cutting, scraping, managing tiny objects or as a weapon.

**Figure 1:**
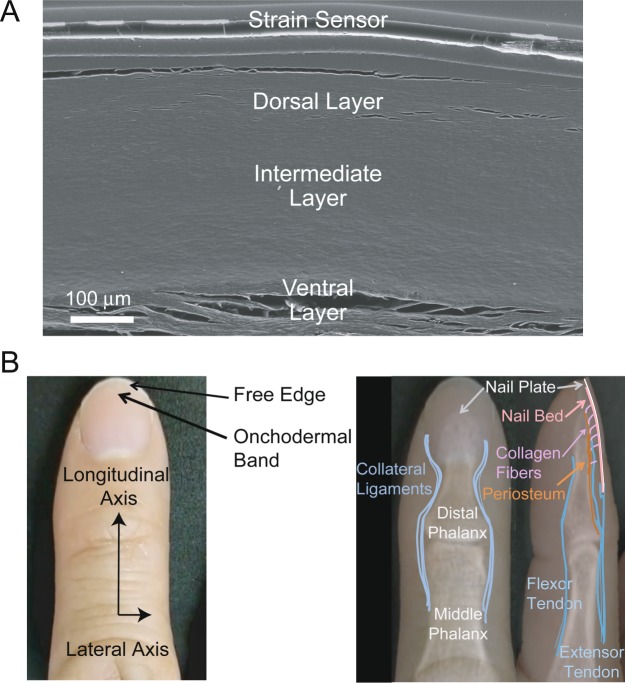
Anatomy of fingernail. (**A**) Scanning electron micrograph of a fingernail cross-section. (**B**) Index finger photograph and X-ray of bone structures with connection schematic.

The ungula or nail plate is the visible part commonly thought of as the fingernail. Three layers of cornified onychocytes make up the ungula. Figure 1A is a scanning electron micrograph of a cross-section through the author’s nail in which the three strata are visible. Onychocytes are anucleated cells which initially arose from the nail matrix or the nail bed^6^. These hardened, dead cells are filled with fibrous, translucent onychokeratin proteins^7^. The dorsal layer provides a hard, smooth outer covering to the thicker, more flexible intermediate layer. The ventral layer is produced by and bound to the nail bed. The entire nail plate is roughly 0.6 mm thick in males and slightly thinner in females but becomes thicker with age regardless of gender^6^. Non-pathological nail plates have a gentle convex transverse curve along the longitudinal axis of the fingertip and typically advance about 0.1 mm per day^8^. Figure 1B contains a confection created by hand registering a photograph and x-ray, both of one of the author’s fingertip. Work in the context of psoriatic arthritis using high-resolution MRI imaging and histology has shown that the nail bed and plate are ruggedly and comprehensively bound to musculoskeletal structures in four ways^9^. First, collagen fibers arise from the periosteum of the distal phalangeal bone, especially the spatulate tuft at the distal end and attach to the underside of the nail bed. Second, the collateral ligaments stabilizing the distal interphalangeal joint extend and connect to the lateral edges of the nail bed. Third, the extensor tendon attaches to the base of the distal phalangeal bone and continues on to envelop the nail root. Finally, fibers of the extensor tendon also continue along the dorsal aspect of the distal phalange and join a thickened periosteum and nail bed. These tendinous and ligamentous attachments transmit tensile stress but not much compressive stress. The deformable fingertip pulp in direct contact with the lateral edges and the distal end of the nail plate transmits compressive stress but not much tensile stress. The anatomical structure and interactions between the nail plate, distal phalangeal bone, musculoskeletal attachments, and fingertip pulp causes nails to deform in complex but repeatable ways during unloaded movement and in interactions with objects.

Humans use their fingers extensively in activities of daily living. Because of this, nails are tempting structures to characterize for insight into these activities. Our primary objective was to evaluate a system to quantify the kinematics of fingernail deformation and characterize finger and hand movement idioms using a wearable sensor. Identifying grip types, activation profile, and maximum grip force can provide clinical features relevant to diagnosing degenerative neurological diseases such as Parkinson’s disease^10,11^. Hand strength has been correlated with general health-related quality of life^12^. Other studies showed that greater hand strength was positively related to cardiac function^13^, central nervous system health^14^, and cognitive performance among people with schizophrenia^15^. Understanding which tasks require maximum grip force or finger pressure and how often repetitive actions occur is necessary to identify and ultimately refactor injury producing workplace activities. Onycholysis, paronychia, and koilonychia are examples of nail disorders that can result from repetitive microtrauma^16^. Various forms of tendinopathy and repetitive strain injuries can result from poorly designed or stressful job activities^17^.

In previous work, several groups have investigated the mechanics of fingertip loading and force transmission to better understand the relationship between workplace tasks and muscle fatigue, pain, onycholysis, and soft tissue injuries (carpal tunnel syndrome, flexor tenosynovitis, etc.).

The most comprehensive fingertip instrumentation effort was carried out by Reid *et al*.^18^ in the context of extravehicular activity glove design for astronauts. They studied blood perfusion, galvanic skin response, longitudinal and transverse fingernail strain and pressure relative to force plate and dynamometer data during a battery of static and dynamic tasks. Fingernail strain during static pressure at various fingertip angles was quantified by Sakai *et al*.^19^. This work produced curves relating fingernail strain in the longitudinal and transverse axes during fingertip compression using three nail-mounted strain gauges.

To build a biomechanical model of the hand the force response of fingertip pulp to repeated compression was studied in quasi-static conditions using cameras and force transducers on a plate during a tapping task^20^. Pulp deformation and recovery after repeated indention cycles was studied by D’Angelo *et al*.^21^, but they did not consider the effects over multiple days and attributed all results to viscoelastic effects. All the above studies provided insights into fingertip dynamics and nail deformation through fingertip interaction with surfaces but were limited to bench activities and hence not able to characterize activity in daily life.

Other authors have explored the possibility of using nail-mounted devices for human-computer interactions. The original work in this area used nail-mounted cameras to capture differences in the hemodynamic state of the fingertip visible through the nail during different types of forces. This photoplethysmographic fingernail sensor was used to construct a wearable mouse that derived horizontal motion, vertical motion, and click behavior from changes in fingertip blood volume via an array of nail-mounted of LEDs and photodetectors^22^. Another example, NailO^23^, prototyped a nail-mounted gestural input surface that fit entirely on the nail. This device used projected capacitance to detect touch events and communicate wirelessly with remote computing devices. This device was also limited to simple directional swipe and click gestures similar to a mouse. Non-existent or occluded input device type controls were presented on a fingernail mounted OLED display in the NailDisplay^24^ project. It included an accelerometer to capture basic swipe gestures. Finally, FingerPad^25^ was a thumb, and index fingernail mounted device which used magnetic tracking to deconvolve the motion of the thumb on the index finger to input digits. To the best of our knowledge, no system has integrated both nail strain and accelerometer information to explore human hand biomechanics and enabled an unconstrained human-computer interaction. Table 1 summarizes the contribution of our system versus previous projects and devices.

**Table 1:** Comparison of related work.

Project	Sensor	Wearable	Grip strength correlation	Behavioral and Biomechanical monitoring	Human-Computer Interface	Ref.
This paper	Strain gauges and accelerometer	Yes	Yes	Yes	Yes	—
EVA Glove	Force sensitive resistor, strain gauge, piezoresistive pressure sensor, laser doppler, humidity sensor, etc.	No	Yes	Yes	No	^18^
Sakai and Shimawaki	Strain gauges	No	Yes	Yes	No	^19^
Serina	LEDs and cameras	No	No	Yes	No	^20^
D’Angelo	Force sensor and laser	No	No	Yes	No	^21^
Mascaro and Asada	LEDs and photodetectors	No	Yes	Yes	Yes	^22^
NailO	Capacitive touch sensor	Yes	No	No	Yes	^23^
NailDisplay	Color OLED display and vibrator	Yes	No	No	Yes	^24^
FingerPad	Magnet	No	No	No	Yes	^25^
Apple Watch	Accelerometer, LEDs and photodetectors	Yes	No	Yes	Yes	

We present a wearable fingernail deformation system containing a strain gauge, electronics, and software. This system characterizes grip, gestures, and more complicated motion idioms. Characteristics of motion idioms such as duration, force, and fluency describe a human subject’s state. The system also functions as a human-computer interface by translating nail deformation signals during finger writing to characters. Finger writing can take place on any convenient surface such as a table, desk, wall, clothing, or subject’s other palm. A finger appropriately configured with a sensor could use gestures to perform computer operations such as scrolling, paging, shrinking or expanding images. While our ultimate goal is to make a wearable device that is entirely on the nail and to develop a platform that has the capability of characterizing various kinds of idioms and gestures, this paper describes a prototype for nail deformation sensing with a wearable device and results of the demonstration of this concept.

## Results

**Figure 2:**
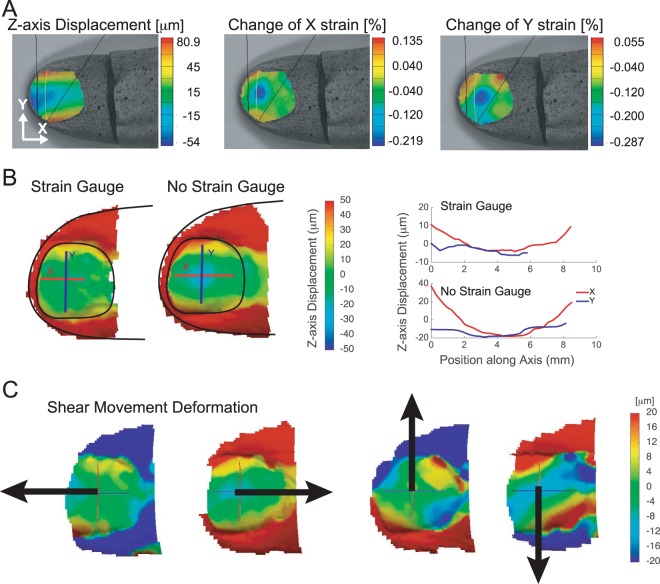
Fingertip surface profile measurements. DIC images of fingernail during normal force test. (**A**) Left to Right: Z-axis displacement, strain along X (center), and strain along Y (right) directions. (**B**) Z-axis displacement and strain profile at five newtons with and without a strain gauge. (**C**) Z-axis displacement during shear forces.

To find optimal locations on the nail for measuring strain forces (see Methods) we initially quantified deformation across the entire nail during normal force using a three-dimensional Digital Image Correlation (DIC) measurement system (see Methods). Figure 2A shows three-dimensional surface profile measurements of a fingertip produced by the DIC technique. When the fingertip pad was pressed against a test surface with normal force, the distal phalangeal bone pulled the center of the nail downwards, and the fingertip pulp moved around the distal phalangeal bone to push up against the nail at the lateral folds, deforming the edges upward. The leftmost image in Figure 2A shows displacement, while strain in the fingernail is shown in the center and right images respectively. The displacement (*dL*) was more than 130 µm (from −54 µm to +80 µm) and the strain change was more than 0.3% (from −0.21% to +0.13%). The tests were repeated to understand the consistency of the DIC measurement and the data remained qualitatively similar. We concluded that a strain sensor attached to fingernail needs to be flexible enough to tolerate the anticipated deformation (130 µm) and sensitive enough to detect strain in the range of ±0.20% (=2000 µɛ). From the DIC measurement results, the positions with the most deformation were across the nail at the top of the lateral nail folds. Figure 2B shows deformation at five newtons of normal force for a nail with and without a strain gauge glued to it. The profile plots on the right show that the maximum Z-axis displacement longitudinally was 40 µm without the strain gauge and 10 µm with it. The displacement in the transverse direction was −7 µm with the strain gauge and −20 µm with it. These results show that when a strain gauge is glued to the nail it is considerably stiffer and less responsive. Figure 2C shows nail deformation during shear movements. In general, as finger pulp is pulled away from the direction of motion, it pulls the forward edge of the nail with it.

### Hardware Stack Description

**Figure 3:**
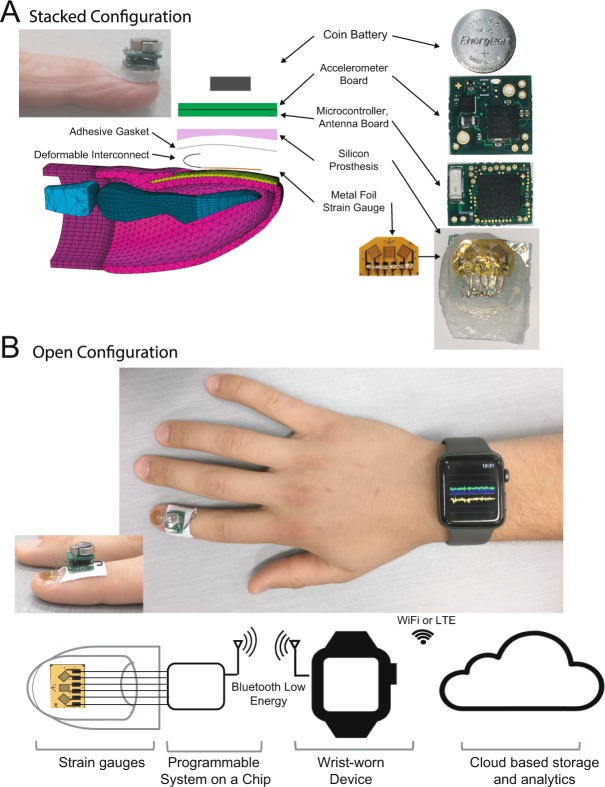
Schematic diagram of the wearable system. (**A**) Electronics with silicone prosthetic on the nail. (**B**) Electronics on the skin with for debugging. Schematics of the fingertip model in panel A have been reproduced with permission by Bucknell Webb.

The DIC results informed our decision to use a metal foil strain gauge as well as providing the intuition that a low modulus prosthetic would absorb the amount of nail deformation likely to be seen during normal activities. We used two configurations during our testing. In the first configuration, the electronics ride on a silicone prosthetic on the nail as shown in Figure 3A. In the second configuration, seen in Figure 3B the electronics were placed on the skin with a gasket leaving the strain gauges visible. This visibility allowed us to monitor and document the position and adhesion of the strain gauges on the nail during testing. Similar to the strain gauges the low modulus silicon prosthetic affected nail deformation. These effects were consistent, and so it was possible to train models specific to the configuration. Subjects were conscious of having the device on their nail, similar to wearing a watch. No subject reported discomfort.

The metal foil strain gauges were rigidly attached to the nail with over the counter cosmetic cyanoacrylate nail glue (Kiss Professional Nail Glue). We used the three gauge 45-degree rosette (SGD-1/350-RY21, Omega Engineering) to capture strain readings at different points along the nail rather than to compute the strain tensor. The center gauge responds to strain in the longitudinal direction only while the outer gauges, oriented at forty-five degrees react to a combination of both transverse and longitudinal strain.

For each subject, we created a low modulus silicon prosthesis using Skin Tite Silicone Prosthetic Builder, (Smooth-On Inc., Macungie, PA, USA). This product is intended for movie special effects and medical prosthetics. The prosthetic adapts a flat upper surface to mount the rigid boards for the electronics to the surface of the curved nail topology below. The prosthesis absorbs nail flexion without undue mechanical loading of the nail which could potentially change its behavior or cause nail problems (e.g., onycholysis). The prosthetic attaches via a gasket to the top of the strain gauge and nail surrounding it. The gasket is a fabric patch coated with silicon glue (Sil-Poxy Silicone Rubber Adhesive, Smooth-On Inc) on top and medical adhesive (Pros-Aide Adhesive, ADM Tronics, Inc.) on the bottom. A deformable interconnect is threaded through the gasket and prosthetic to connect the strain gauges with and board 1 (see Fig. 3A). Two electronics boards sit on top of the prosthesis. Board 1 contains a 4XX8 BLE 4.2 family programmable system on a chip (PSoC, Cypress Semiconductor, San Jose, CA). This chip includes an ARM Cortex-M0 CPU and Bluetooth Low Energy (BLE) radio and subsystem. Board 2 contains a 3-axis accelerometer (ADXL335 IMU Analog Devices, Norwood MA) and a coin battery holder. At the top of the stack a 1.55 volt, 80 mA, silver oxide, high drain battery (Energizer 393/309) fits into the battery holder. On the PSoC, strain and acceleration values are captured at 50 Hz, analog to digital conversion performed, and the resulting values joined with an oscillator value. The oscillator runs at 32.768 kHz, and values reset every time the PSoC initializes. This value provides a way to determine if observations are lost, or the PSoC has been interrupted or restarted and is also used to space the incoming data properly in time sequence since it will be received in clumps and delayed non-deterministically by the Bluetooth protocol. The X, Y, and Z accelerometer values, and right, center, and left strain gauge values are all digital versions of continuous values after ADC processing. The strain values correspond to the strain gauge resistor divider voltage amplified via a two-stage inverting amplifier op-amp circuit. The distance from fingernail to wrist is on the order of six inches which makes it too far for near field communication (NFC), but well within the range of Bluetooth low energy (BLE). Since it is known that the distance from the device to watch will always be in this range, power settings can be reduced. For testing and debugging, we used a USB dongle plugged into a laptop running MATLAB scripts to plot and record the data. For naturalistic task testing, we used a Series 3 Apple watch (Apple Inc, Cupertino CA, USA) worn on the wrist to communicate with the PSoC to receive the data tracks. The raw data was sent via a paired iPhone on to cloud-based machines for retention, analysis and model training.

### Nail strain measurement under basic forces

**Figure 4:**
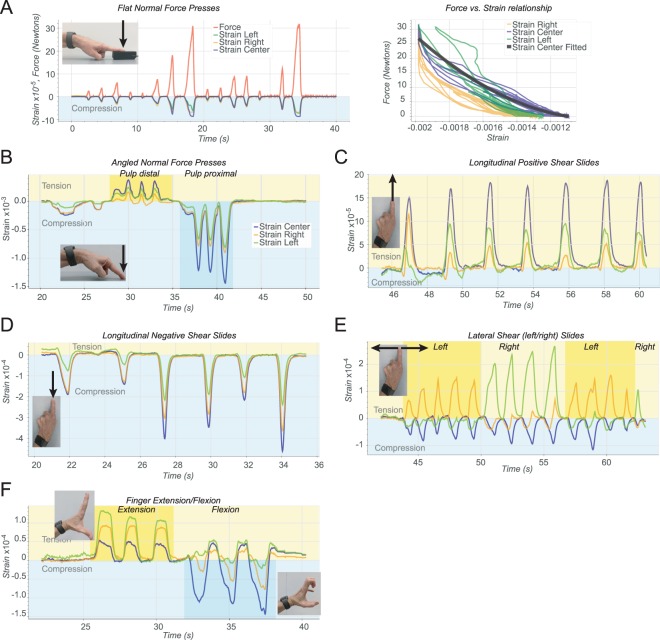
Examples of strain response data. (**A**) Strain response data for a series of flat normal force presses against the surface of a dynamometer (left) and strain vs. force curves for these same actions (right). (**B**) Strain response for a series of normal force, forty-five-degree angle finger presses on a glass surface with the finger pulp first distally and then proximally displaced (see Supplementary Movie 1). (**C**) Responses when the finger is pushed away and the onychodermal band pulls down on the distal end of the nail imparting bending (tensile) stress. (**D**) Strain responses for a series of sliding motions toward from the subject under a normal force on a glass surface producing compressive stress. (**E**) Strain responses to a series of left and right lateral movements under a normal force on the glass surface. (**F**) A set of three finger extensions followed by three flexion movements.

**Figure 5:**
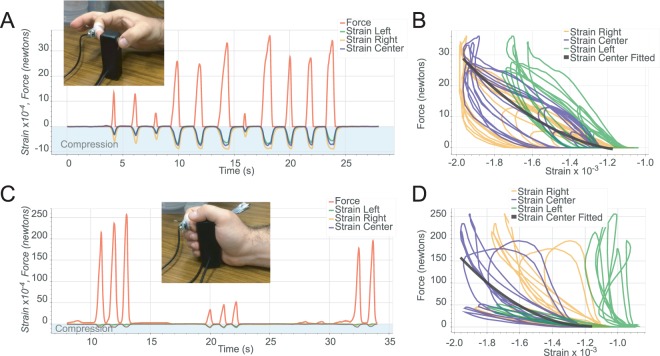
Examples of strain responses during precision and power grips. (**A**) Strain and force responses during a set of precision pinch grips. (**B**) Strain versus force responses during precision pinch. (**C**) Responses for a power grip session. (**D**) Strain versus force responses during power grip.

We gathered information about how the nail deforms under strain responses during some simple actions before investigating less restricted tasks. In all cases, the device was positioned on the index finger (either left or right, but independent of the dominant side). Figure 4 shows example responses for a representative subject to presses against a flat surface with a perpendicular force with a flat finger, finger oriented forty-five-degree angle, sliding motions, and lateral movements respectively. Next, Figure 5 shows examples of responses during power and precision grips.

On the left in Figure 4A strain response data for a series of normal force presses against the surface of a dynamometer (NEULOG NUL-237, resolution: 0.1 N) and USB module (NEULOG USB-200) as shown in the inset photograph, is plotted with the corresponding force values. On the right in Figure 4A are the strain vs. force curves for these same actions. The signal saturates for the right and center gauges meaning that at a certain point more force was applied but the nail did not continue to deform. Fitting a polynomial to the center strain response gave an intrasession r-squared 0.97 for this subject and an intersession r-squared of 0.84.

Figure 4B shows the strain response for a series of forty-five-degree angle finger presses on a glass surface with the finger pulp first distally and then proximally displaced. There has been some discrepancy in previous work^19^ concerning the polarity of strain in response to normal force. We demonstrate here that the disposition of the finger pulp from distal to proximal relative to the distal phalangeal bone can switch the polarity of strain from compressive to tensile during normal force. Supplementary Movie 1 is a recording of this and the next four tests.

Figure 4C shows strain responses for a series of sliding motions away from the subject under a normal force where the onychodermal band pulls down on the distal end of the nail and imparts bending (tensile) stress. In these movements, we see that since the center strain gauge is aligned to respond to changes along the longitudinal axis and the gauges at the lateral edges are at a forty-five-degree angle they only produce half the response. Figure 4D shows the strain responses for a series of sliding motions toward the subject under a normal force on a glass surface. These produce longitudinal shear forces which displace the finger pulp distally, and the free edge of the nail is pushed upwards, compressing (or straightening) the nail in the longitudinal axis.

Figure 4E shows strain responses to a series of left and right lateral movements under a normal force on the glass surface. The first five movements are left to right, the second four are right to left, and the final four are left to right. In each case, the leading, lateral side of the fingernail and the center are under compressive stress, and the trailing edge is under tensile stress during these movements.

Figure 4F shows a set of three finger extension followed by three flexion movements. In the case of extensions, the extensor digitorum muscle contracts to put tension on the extensor digitorum tendon which terminates on the base of the distal phalangeal bone and attaches to the proximal end of the nail bed. Tension from this tendon on the proximal end of the nail bed anchored by central and distal connections to the distal phalangeal bone results in tensile strain on the nail plate as measured in this test. These examples illustrate structures other than finger pulp acting on the nail. In this case, there was no contact with any external object, and only musculoskeletal connections were involved.

These tests demonstrate that the sensor system can capture the orientation, direction, and force of interactions at the fingertip. Due to the anatomical links between the fingernail and the musculoskeletal system, it is also possible to capture differential forces produced by movements that have no object interaction. We showed this in Figure 4F with a set of three finger extensions followed by three flexion movements.

Next, we demonstrate the nail sensor characterizing grip related actions. Figure 5A shows the strain and force responses during a set of precision pinch grips. In Figure 5B the strain versus force responses are plotted. It should be noted that the center strain gauge did not saturate even at maximum grip force.

Figure 5C shows the responses for a power grip session. In a precision grip all the grip force is transmitted through the index finger pad, while in a power grip, the entire length of all the fingers, and the palm contribute to the grip force. In Fig. 5D none of the strain gauges saturated and the force generated by the power grip was almost an order of magnitude higher than the precision pinch. With a strain gauge on a single finger, we were unable to predict grip type. Consequently, we could not predict the overall grip force without knowing the grip type.

### Nail-deformation sensor for dexterous movements and human-computer interaction

**Figure 6:**
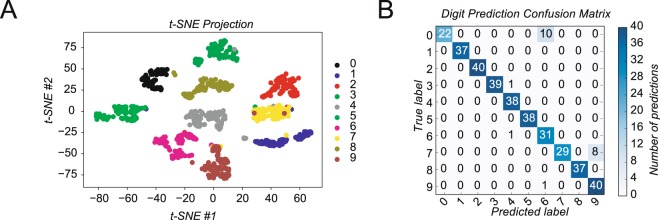
Detection of finger movement idioms. (**A**) T-SNE visualization showing separation of strain signals color coded according to their true values. (**B**) Confusion matrix of correctly and incorrectly predicted finger digits for the test set. The absolute number of trials is reported.

To explore using nail strain measurements as a human-computer interface and demonstrate identifying subtle finger movement idioms, we attempted to predict digits ‘written’ with a fingertip on the screen of a tablet (see Methods, see Supplementary Movie 2). Subjects recorded digits during four sessions on four different days. We used a new strain gauge for each test to eliminate the possibility of damage during removal effecting subsequent tests. Two sessions were used for training and the remaining two for test and validation. Each digit was represented as a time series of strain values (see Supplementary Fig. 1). As a demonstration of how unique this data was for each digit, we created an unsupervised t-distributed stochastic neighbor embedding (t-SNE) plot of the data for all four sessions from a single subject. The purpose of t-SNE analysis is to show that the continuous high-dimensional time series can be reduced to a lower dimensional space in which the digits are clearly separated. Indeed, Figure 6A shows the results when the t-SNE technique has been applied to reduce the data to two dimensions. In this representation, each data point is colored according to its actual digit label. There are well-separated clusters for each digit.

To predict digits from strain voltage readings we used a 1-nearest neighbor algorithm with the Procrustes distance as the distance metric. The generalized Procrustes technique overcame the changes we observed in nail deformation response over time and find the optimal superposition between shapes. Using the first two sessions for training and the second two for testing we achieved an accuracy of 98.7% for this subject. Figure 6B contains the full confusion matrix. We observed that over several sessions of finger writing, subject’s fingers became less responsive. We suspect that this was from loosened connections within the fingertip, possibly the anterior tendon but we are unable to prove this.

**Figure 7:**
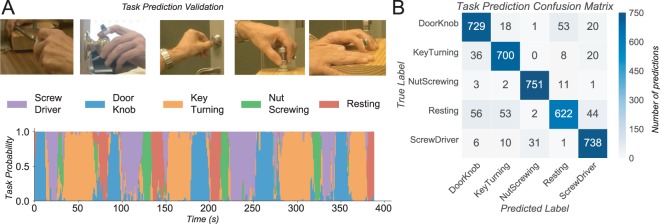
Detection of hand activities. (**A**) Time series of probability activations corresponding to five actions: using a screwdriver, turning a doorknob, turning a key, screwing and unscrewing a nut, and rest. (**B**) Confusion matrix of correctly and incorrectly predicted hand activities for the test set. The absolute number of trials is reported.

To understand other behaviors that might be identifiable from nail deformation but not from wrist-worn devices we captured data for turning a key, turning a doorknob, screwing and unscrewing a nut, using a screwdriver, and rest. We collected three sessions of labeled training data consisting of one to three minutes of each task on three different days. When the first two sessions were used as training data and the third for testing, we achieved an accuracy of 91%. Figure 7A shows a plot of the task probability prediction for each of the activities during the validation session while Figure 7B shows the complete confusion matrix for each window prediction. During an additional trial containing all tasks in a random sequence, strain and video data were collected. Supplementary Movie 3 contrasts the predictions of the continuous activity detection models (for each 1.5 s window) with the actual performance of those tasks.

Finally, we explored the ability to capture data during activities which produced large nail deformations and required strenuous grips without interfering with these actions. As a proof of concept, we monitored the signal during a series of baseball grips and simulated pitches (see Supplementary Movie 4). We reliably received qualitatively specific signals for each grip. These biomechanical insights into finger forces during specialized, highly energetic idioms were recorded without inhibiting the subject’s ability to throw the ball or damaging the sensor.

## Discussion

In this paper, we demonstrated the first of its kind prototype wearable system to capture and characterize fingernail deformation forces. The system is small enough to be worn on a finger, does not interfere with tactile sensing or haptic perception, and is sturdy enough to wear while throwing a baseball. Fingernail deformation forces were used to identify digits written with the fingertip as an example of characterizing subtle finger movements. Finger writing is a simple demonstration of the system as a human-computer interface. We also quantified the relationship between deformation and various grip types and grip force.

Since we used rigid printed circuit boards for the prototype, future hardware improvements will focus on shrinking the electronics using wafer-level chip scale packaging, switching to a flexible substrate and reducing power by changing to micrometer scale. Replacing the large metal foil strain gauge package with tiny silicon strain gauges will allow the nail to move more freely. Packaging and installation present many challenges, as the system should ultimately be waterproof and easy to install and remove. Capturing phenomenology during disease states with known correlations to changes in grip force is a priority. Finally, identifying and quantifying injury producing motions for immediate feedback to the wearer is a goal.

## Methods

### Data collection

All experiments were performed in accordance with institutional guidelines and regulations. The experimental protocol was approved by the IBM Research Institutional Review Board, at the T.J. Watson Research Center, Yorktown Heights N.Y., USA. All participants gave their informed consent to take part in the experiments. Four subjects were involved in finger pressures under basic forces. Three subjects participated in the finger writing tasks and four subjects in the hand actions experiment. One experienced subject executed various baseball pitch grips.

### Measurements of nail forces

Stress is defined as the force acting on a cross-sectional area of a deformable object. Strain is the response to stresses applied to a deformable object, usually defined as the amount of deformation in the direction of the stress divided by the initial length of the object, resulting in a unitless number. Strain (*ɛ*) is defined as $\varepsilon ={dL}/L$ where *L* is the length of the object. In a simple, homogeneous material object, stress and strain have a linear relationship in their elastic range obeying Hooke’s law. The fingertip is not a homogeneous object but is composed of many elements (bone, tendon, ligament, nail, pulp) with different elastic properties. The sensitivity of a strain gauge, known as the gauge factor (*GF*) is defined as the ratio of change in electrical resistance to the strain ${GF}=({dR}/R)/({dL}/L)=({dR}/R)/\varepsilon$ where *R* is the electrical resistance of strain gauge. The gauge factor of a typical metal foil strain gauge ranges between 2 to 6 and strain up to at least 10% can be measured. For example, a strain gauge with a gauge factor *GF* = 2.1 will exhibit a change in electrical resistance of 2.1 × (2000 × 10^−6^) = 0.41%, to support strain of 2000 µɛ. By applying strain gauges to the nail plate, we measure changes in resistance caused by nail plate strain. An additional consideration when using metal foil strain gauges is the amount of current required. In general silicon strain gauges draw less current but are more delicate and require additional packaging invention.

### Quantification of nail deformation

Nail strain forces were quantified as deformation across the entire nail during normal force using a three-dimensional Digital Image Correlation (DIC) measurement system (Aramis − 3D Motion and Deformation Sensor, GOM). This system uses two cameras in a stereoscopic arrangement with a known distance to the object to calculate the position of each surface point. Finding the maximum correlation between the pixel values of small regions on the surface of the object in two different images results in a set of transformation parameters. These transformation parameters are then used to compute deformation and displacement for each region. Strain can be computed from the transformation parameters and changes in deformation.

### Finger idioms

Subjects recorded digits during four sessions on four different days. A new strain gauge was used for each test to eliminate the possibility of damage during the previous removal having an effect on subsequent tests. During each session, up to forty instances of each digit were recorded. An app was used (FieldNotes) to capture which digit was written and when to provide labels for the training data. This labeled data was separated into time series snippets containing the analog-to-digital (A2D) voltage values returned for the three strain gauges while each digit was written. A bandpass filter (from 0.25 Hz to 3 Hz) was applied to the data and the absolute value computed and summed back into the original data. This signal was used to separate each digit in the time series (see Supplementary Fig. 1). The strain data for each digit was padded to form a three by three hundred element matrix, and the first twenty principle components were computed and passed to the t-SNE algorithm. Each data point was colored according to its true digit label.

### Object manipulation

In order to capture key and doorknob turning, nut screwing and unscrewing, and using a screwdriver, one session of labeled training data consisting of six minutes of each task. A final session containing all four tasks (in addition to rest) in random sequences was collected, and video was collected. In this experiment, both strain and accelerometer data were used. All data tracks were resampled to 50 Hz and bandpass filtered (from 0.25 Hz to 15 Hz) to remove the offset and high-frequency noise. Each sample was a matrix of six tracks (three strain and three accelerometer) and seventy-five elements corresponding to a 2-second window that was then moved by 0.25 seconds to form the next sample and was labeled by task. The classification model was a neural network with a hybrid CNN/LSTM architecture^26^. An architecture diagram is included in the Supplementary Figure 2 and consists of one Convolutional layer, a max pool layer, an LSTM, two dense layers and a soft-max layer. All layers are fully connected. The inputs (size 6 × 96) are first processed by a 1D convolutional layer with 100 convolutional filters (kernel size = 5, stride length = 1, linear activation function) followed by a max-pooling layer (size 2) to reduce feature representation from 96 to 48. Convolution is done across time and its resulting feature maps are fed to an LSTM (150 units, linear activation function), then a sequence of two dense layers (100 units and 5 units, linear activation function). Finally, the soft-max output layer computes probability activations over our 5 action states. The model was trained to minimize the cross-entropy loss function using the Adam optimization algorithm. Regularization was done by means of dropout (30% rate before the Dense layers) to reduce overfitting. All the code was developed in python using the Keras library.

## Electronic supplementary material

Supplemental Information

SupplementaryMovie1

SupplementaryMovie2

SupplementaryMovie3

SupplementaryMovie4
