# Supplementary material for: Wearable Nail Deformation Sensing for Behavioral and Biomechanical Monitoring and Human-Computer Interaction
Source: Sci Rep. 2018 Dec 21;8:18031. doi: 10.1038/s41598-018-36834-x (PMC6303398; doi:10.1038/s41598-018-36834-x)
Supplement: Supplementary file 1 — Supplemental Information [file 41598_2018_36834_MOESM1_ESM.docx]

**Supplemental Information**

**Wearable Nail Deformation Sensing for Behavioral and Biomechanical Monitoring and Human-Computer Interaction**

**Katsuyuki Sakuma, Avner Abrami, Gaddi Blumrosen, Stanislav Lukashov, Rajeev Narayanan, Joseph W. Ligman, Vittorio Caggiano, Stephen J. Heisig**


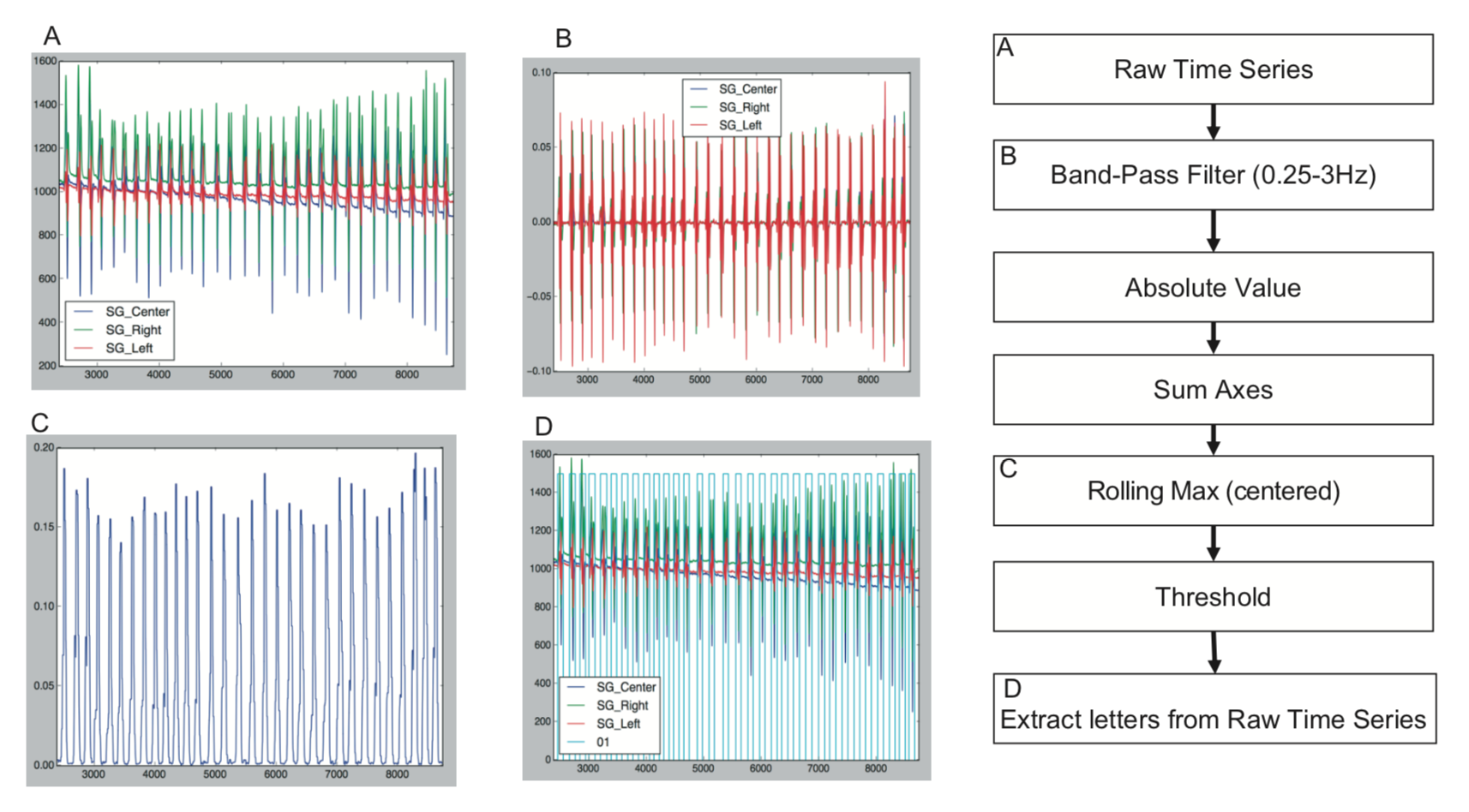


**Supplementary Figure 1 – Pipeline to extract digits from continuous strain signals.**


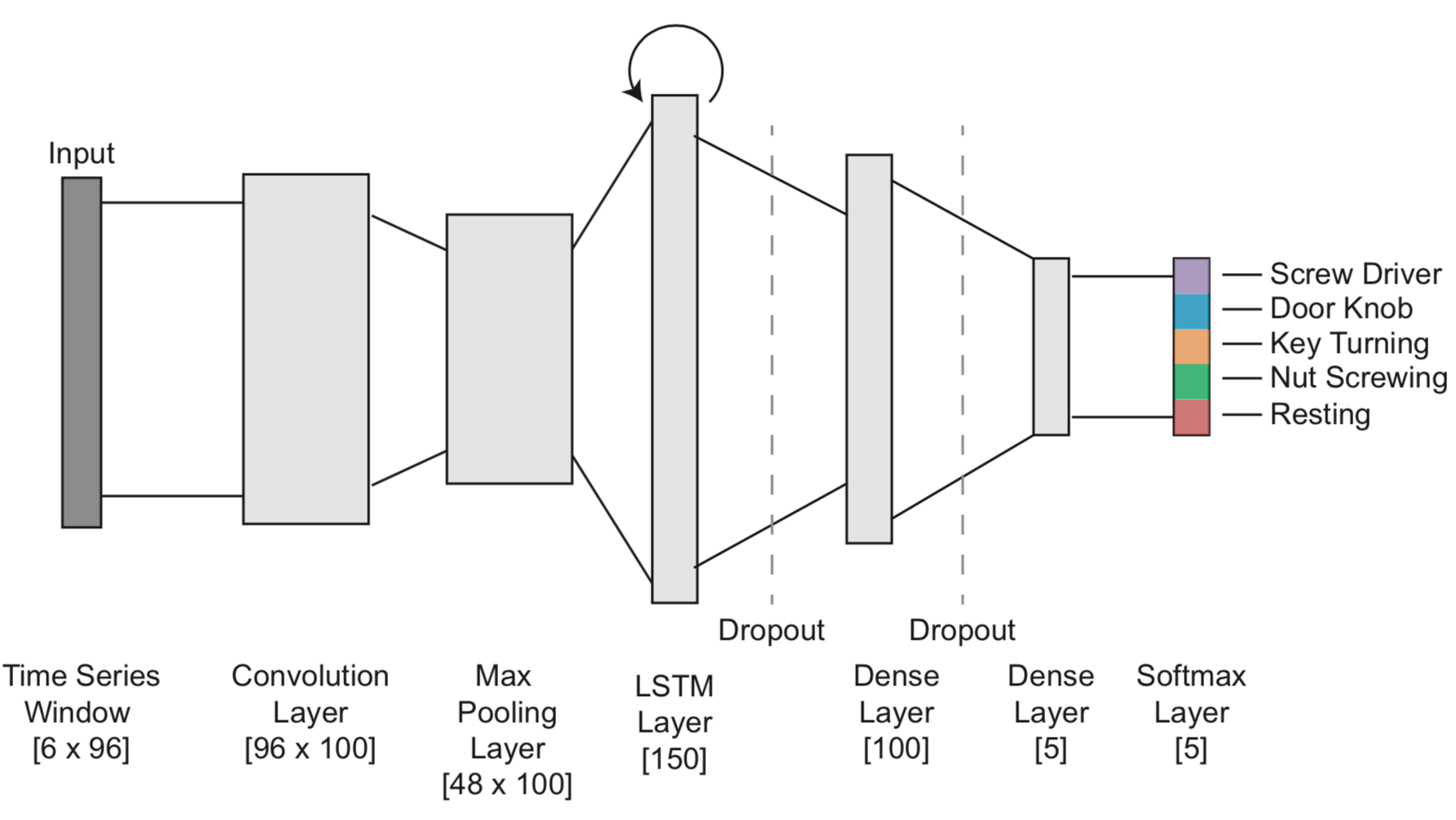


**Supplementary Figure 2 – Neural network architecture to predict hand actions.**

**Supplementary Movie 1 – Strain signal waveforms during basic finger movements.**

**Supplementary Movie 2 – Strain signal waveforms during digit writing.**

**Supplementary Movie 3 – Task predictions while performing hand tasks.**

**Supplementary Movie 4 – Strain signal waveforms during baseball grips.**
